# Treatment of Knee Osteoarthritis with Intraarticular Umbilical Cord-Derived Wharton’s Jelly: A Case Report

**DOI:** 10.3390/ph14090883

**Published:** 2021-08-31

**Authors:** Ashim Gupta, Hugo C. Rodriguez, Anish G. Potty, Howard J. Levy, Saadiq F. El-Amin III

**Affiliations:** 1 BioIntegrate, Lawrenceville, GA 30043, USA; hlevymd@hotmail.com (H.J.L.); dr.saadiqelamin@gmail.com (S.F.E.-A.III); 2 Future Biologics, Lawrenceville, GA 30043, USA; 3 South Texas Orthopedic Research Institute (STORI Inc.), Laredo, TX 78045, USA; anishpotty@gmail.com; 4 Veterans in Pain, Valencia, CA 91354, USA; 5 Holly Cross Orthopaedic Institute, Fort Lauderdale, FL 33334, USA; hcrodrig2112@gmail.com; 6 Laredo Sports Medicine Clinic, Laredo, TX 78041, USA; 7 Department of Orthopaedic Surgery, Lenox Hill Hospital, Northwell Health, New York, NY 10075, USA; 8 El-Amin Orthopaedic and Sports Medicine Institute, Lawrenceville, GA 30043, USA

**Keywords:** umbilical cord, Wharton’s jelly, knee osteoarthritis, regenerative medicine, biologics, exosomes, extracellular vesicles, growth factors, cytokines, hyaluronic acid

## Abstract

We present the case of a 27-year-old male with grade II knee osteoarthritis (OA) that was intraarticularly injected with a 2 mL umbilical cord-derived Wharton’s jelly (UC-derived WJ) formulation. The patients’ baseline radiographs were taken and baseline numeric pain rating scale (NPRS), knee injury and osteoarthritis outcome score (KOOS), 7-point Likert scale, and a 36-item short form survey (SF-36) were recorded. The NPRS was re-recorded immediately after the injection, and at 24 h, 48 h, 1 week, 6 weeks, and at 3 months follow-up post-injection. The KOOS and 7-point Likert scale was re-recorded at the patients’ 1week, 6 week, and 3month follow-up, and SF-36 was re-recorded at 3 months. A final set of X-rays were also performed at 3 months follow-up post-injection. No adverse effects from the injection were reported over the duration of the study. No significant difference nor progression in OA on X-rays compared to baseline was observed. NPRS decreased by 50% and the 7-point Likert scale increased to Extremely Satisfied. KOOS increased overall by 10% and the SF-36 overall change was 25%. These results indicate the potential application of UC-derived WJ in the treatment of knee OA. Larger, long term, non-randomized and randomized control trials are warranted to adequately assess the safety and efficacy of UC-derived WJ and ultimate clinical use.

## 1. Introduction

Osteoarthritis (OA) is the most common joint disorder in the United States (US), affecting over 30 million adults aged 25–74 years [1]. The pathophysiology of OA centers around inflammation and the decrease in vascularization in the degeneration of articular cartilage, resulting in significant pain and decrease in function. OA commonly affects larger weight-bearing joints such as the hips and knees, with the most prevalent being the knee joint [2]. The goal of knee OA treatment is to decrease pain, restore joint function, correct morphological or alignment defects, and improve quality of life [3]. The current treatment options used in clinical practice to manage knee OA, such as non-steroidal anti-inflammatory drugs (NSAIDs), corticosteroids, viscosupplementation, and narcotics have shown variable and limited clinical benefits, but have potential adverse effects [3]. When these conservative measures fail, total knee replacement (TKR) is usually recommended. TKR also has complications, potentially requiring revision surgeries. For these reasons, the field of regenerative medicine and the use of biologics, including umbilical cord-derived Wharton’s jelly (UC-derived WJ) has gained popularity [3]. Our recently published study concluded that UC-derived WJ extract contained large quantities of regenerative factors, including growth factors (GFs), cytokines (CKs), hyaluronic acid (HA, and extracellular vesicles (EVs) compared with other biologics [3]. The presence of these factors gives UC-derived WJ tremendous potential in treating musculoskeletal conditions.

We present the first case where a patient with grade II (on the Kellgren–Lawrence scale) OA was treated with an intra-articular injection (IAI) of UC-derived WJ.

## 2. Case

### 2.1. Umbilical Cord-Derived Wharton’s Jelly

In our recently published study, we described preparation and characterization of this UC-derived WJ formulation [3]. Briefly, following standards established by the U.S. Food and Drug Administration (FDA) and the American Association of Tissue Banks, human umbilical cords were obtained from consenting caesarian section (c-section) donors. These donors underwent comprehensive social, medical, and blood testing before donation. Infectious disease testing was performed at an independent certified laboratory in accordance with the Clinical Laboratory Improvement Amendments of 1988 (CLIA) and 42 CFR part 493 and the FDA [3]. The procured cord was then processed to isolate Wharton’s jelly by rinsing the cord with saline followed by removal of blood vessels, while preserving its structural integrity [3]. The processing was performed aseptically, and the process did not involve the use of any digestive enzymes or cryoprotectants [3]. This formulation was prepared according to the minimal manipulation criteria defined by the FDA, did not include any combination products, and was not intended to depend on the metabolic activity of living cells [3]. This formulation was tested for sterility at an independent CLIA accredited laboratory, Eurofins VRL Laboratories (Centennial, CO, USA), under United States Pharmacopeia Chapter 71—Sterility Testing guidelines [3], prior to use for this case report.

### 2.2. Case Presentation

**Figure 1:**
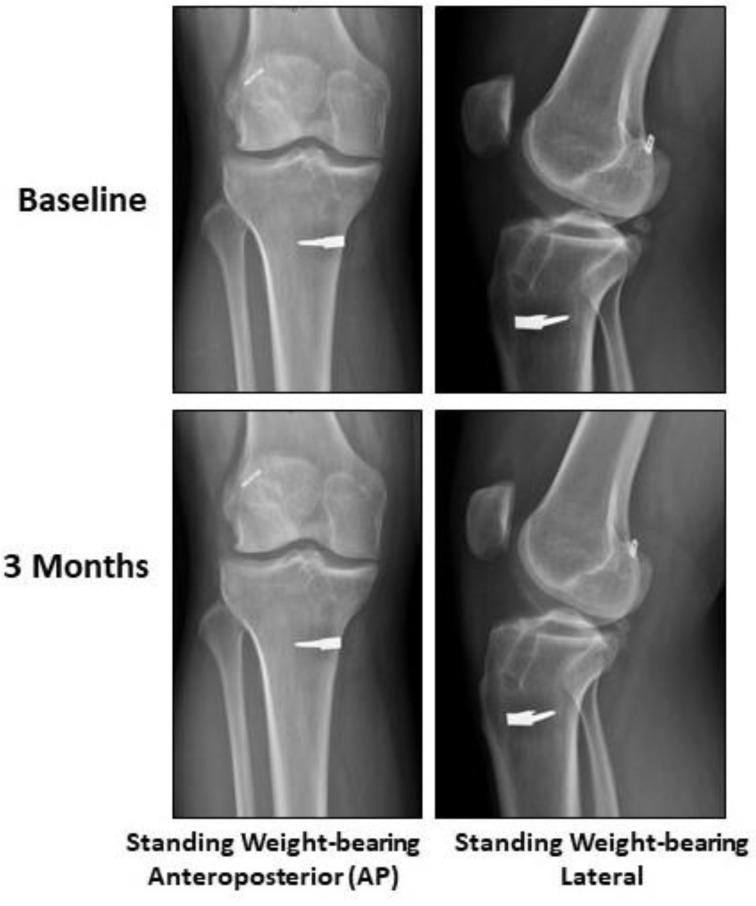
Plain Radiography. No significant difference nor progression in OA via X-Rays was observed.

A 27-year-old male with a medical history of right knee anterior cruciate ligament (ACL) reconstruction presented with pain associated with prolonged weight-bearing and activity. A physical exam revealed normal active range of motion (ROM), mild crepitus, and normal passive ROM. Special tests revealed a (+) McMurray’s, (+) Anterior Drawer, (+) Pivot Shift, and a (+) Lachman with no other abnormalities. Baseline X-rays (weight-bearing AP and lateral views) of the right knee showed evidence of grade II OA with lateral osteophytes, a lateral joint space of 0.92 and a medial joint space of 0.69, valgus alignment, and evidence of prior ACL reconstruction (Figure 1).The subject’s baseline numeric pain rating scale (NPRS), knee injury and osteoarthritis outcome score (KOOS), 7-point Likert scale, and 36-item short form survey (SF-36) were recorded. The subject’s right knee was then injected intra-articularly through the anteromedial portal with 2 mL of UC-derived WJ formulation.

The NPRS was re-recorded immediately after the injection, and at 24 h, 48 h, 1 week, 6 weeks, and at 3 months follow-up post-injection. Their KOOS and 7-point Likert scale was re-recorded at their 1 week, 6 week, and 3 month follow-up, and SF-36 at 3 months follow-up post-injection. A final set of X-rays were also performed at 3 months follow-up post-injection.

No adverse or severe adverse effects from the injection were reported over the duration of the study. No significant difference nor progression in OA via X-rays compared to baseline was observed (Figure 1).

**Figure 2:**
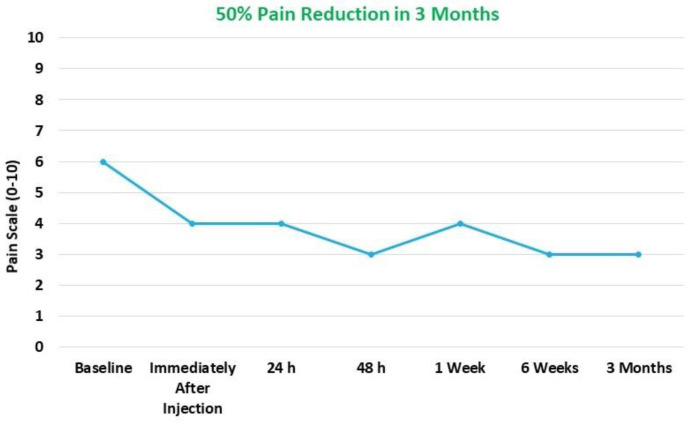
Numeric Pain Rating Scale (NPRS); 50% pain reduction over a period of 3 months compared to baseline.

NPRS showed 50% reduction in pain at 3 months compared to the baseline (Figure 2).

**Figure 3:**
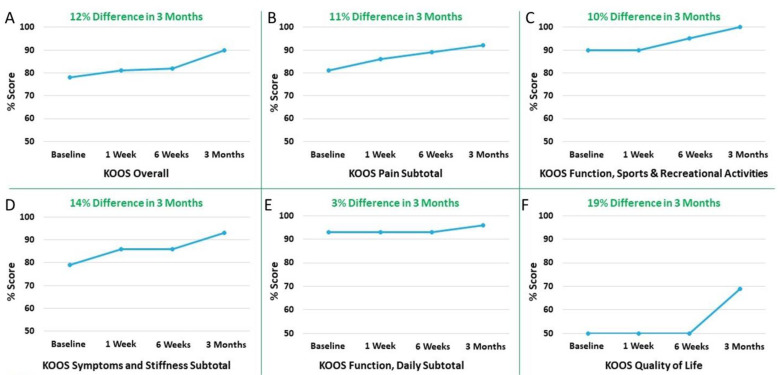
Knee Injury and Osteoarthritis Outcomes Score (KOOS): (**A**) KOOS Overall; (**B**) KOOS Pain Subtotal; (**C**) KOOS Function, Sports, and Recreational Activities; (**D**) KOOS Symptoms and Stiffness Subtotal; (**E**) KOOS Function, Daily Subtotal; and (**F**) KOOS Quality of Life. 12% difference (improvement) was observed over a period of 3 months compared to baseline in overall KOOS; 11%, 10%, 14%, 3%, and 19% difference (improvement) was observed over a period of 3 months compared to baseline in individual KOOS subscales including Pain Subtotal; Function, Sports, and Recreational Activities; Symptoms and Stiffness Subtotal; Function, Daily Subtotal; and Quality of Life, respectively.

Overall KOOS increased by over 10% with the subsets of Symptoms and Stiffness; Pain Subtotal; Function, Sports, and Recreational Activities; and Quality of Life increased by 14%, 11%, 10%, and 19%, respectively, at 3 months compared to the baseline (Figure 3).

**Figure 4:**
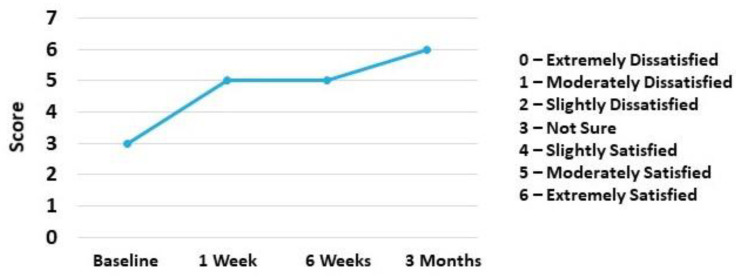
7-point Likert Scale. Patient was “extremely satisfied” at end of 3 months compared to “not sure” at baseline.

Patients’ satisfaction measured via 7-point Likert scale increased from Not Sure at baseline to Extremely Satisfied at 3 months (Figure 4).

**Figure 5:**
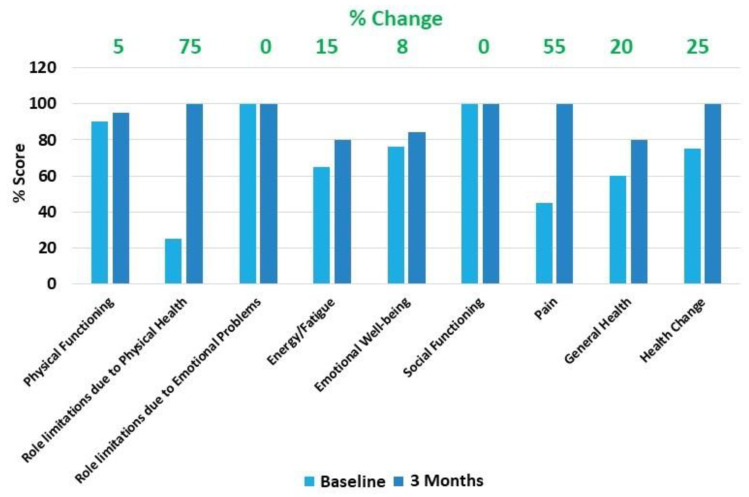
36-Item Short Form Survey (SF-36). SF-36 showed 25% improvement in overall Health change at 3 months follow-up compared to baseline. The individual SF-36 subscales analysis showed improvements in Physical Functioning (5%), Role limitations due to Physical Health (75%), Energy/Fatigue (15%), Emotional Well-being (8%), Pain (55%), and General Health (20%) at 3 months follow-up compared to baseline. The patient did not report any concerns with SF-36 subscales of Role limitation due to Emotional Problems and Social Functioning at baseline.

The SF-36 showed 25% improvement in overall Health change at 3 months follow-up compared to baseline. The individual SF-36 subscales analysis showed improvements in Physical Functioning (5%), Role limitations due to Physical Health (75%), Energy/Fatigue (15%), Emotional Well-being (8%), Pain (55%), and General Health (20%) at 3 months follow-up compared to baseline. The patient did not report any concerns with the SF-36 subscales of role limitation due to emotional problems and social functioning at baseline (Figure 5).

## 3. Discussion

OA is highly prevalent in the population, and current treatment options have historically been limited, specifically for patients suffering from grade II/III knee OA. Additionally, the available treatment options only focus on treating the associated pain and inflammation with little or no effect on the underlying pathophysiology. Although there is no clear etiology for knee OA, many factors such as age, gender, weight, trauma, and mechanical damage can all contribute [4]. Regardless of the exact etiology, the pathophysiology seems to be linked with chronic inflammation, leading to chondrocyte apoptosis and subsequent lesions [5]. In addition, the associated decreases in subchondral blood vessels and of the long-chain HA needed to generate chondrocytes all result in further cartilage damage [6,7]. This overall process results in joint stiffness, pain, decreased function, and subsequent poor quality of life. Currently, the most common medications include NSAIDs, acetaminophen, and opioids, all of which only address the associated symptoms and have a vast side effect profile (gastritis, nephritis, liver dysfunction, central nervous system (CNS) depression, and/or dependence) [8]. Other options, such as IAIs with corticosteroids and viscosupplementation (hyaluronic acid) are used in abundance. Intraarticular corticosteroid and HA injections, although clinically used, are currently not recommended by the American Academy of Orthopedic Surgeons due to conflicting and low-level evidence [9,10]. Cortisone injections have also been shown to have notable side effects, such as joint swelling, stiffness, and arthralgia, as well as being associated with OA progression [8]. Corticosteroids have also been shown to be chondrotoxic and result in cartilage volume loss, especially when combined with local anesthetics [8]. The perioperative use of corticosteroids has also been associated with higher rates of postoperative infection in patients undergoing TKA [11].

For the reasons listed above, attention has been directed towards regenerative therapies including mesenchymal stem cells (MSCs), with over 144 clinical trials investigating their effects on OA [7]. The beneficial effects of MSCs stem from their ability to secrete high levels of GFs, CKs, and EVs, which help establish a regenerative microenvironment. GFs are known to promote cell proliferation, growth, and differentiation, while CKs help to regulate inflammation, cell division, differentiation, and regeneration [12]. Although there are various MSC options, not all are created equal [13]. Autologous MSCs from bone marrow or adipose tissue have a long history of use but seem to be less favorable in terms of potency as compared to allogenic UC-WJ [3].

An allogenic UC-derived WJ formulation was used in this study in order to have a high level of these bioactive factors, such as Insulin-like growth factor binding proteins -1,2,3,4, and 6; transforming growth factor alpha; platelet-derived growth factor-AA; hepatocyte GF; and fibroblast GF, as well as various CKs, such as macrophage colony-stimulating factor, tissue inhibitor of metalloproteinases 1 and 2, interleukin 1 receptor antagonist, and intercellular adhesion molecule-1 [3]. In addition, this formulation has hyaluronic acid and extracellular vesicles that help further promote a regenerative microenvironment [3].

In order to gauge the clinical results of the UC-derived WJ formulation for knee OA, the NPRS, 7-point Likert scales, SF-36, and KOOS score were utilized. These sets of surveys, forms, and scores were used due to their history of being valid, reliable, and responsive outcome measures [14,15,16]. Results from this case showed that a 2 mL intraarticular injection of UC-derived WJ formulation was safe and showed the potential to help improve pain and function associated with knee OA. There was a 50% reduction in pain, and over 10% improvement in the overall KOOS score, both of which are considered clinically significant [16]. These results correlated with the improvements in the 7-point Likert scale and with the 25% improvement in SF-36, indicating the mitigating potential of UC-derived WJ for knee OA.

## 4. Conclusions

In conclusion, this case presents a successful example of the use of the intra-articular administered UC-derived WJ for the alleviation of symptoms related to knee OA. UC-derived WJ has potential in mitigating the progression and the symptoms of OA. Larger long-term, non-randomized and randomized control trials are warranted to adequately assess the safety and efficacy of UC-derived WJ and its ultimate clinical use [17,18].

## Data Availability

Data is contained within the article.

## References

[1] Cisternas M.G., Murphy L., Sacks J.J., Solomon D.H., Pasta D.J., Helmick C.G. (2016). Alternative Methods for Defining Osteoarthritis and the Impact on Estimating Prevalence in a US Population-Based Survey. Arthritis Care Res..

[2] Losina E., Thornhill T.S., Rome B.N., Wright J., Katz J.N. (2012). The dramatic increase in total knee replacement utilization rates in the United States cannot be fully explained by brought in population size and the obesity epidemic. J. Bone Joint Surg. Am..

[3] Gupta A., El-Amin S.F., Levy H.J., Sze-Tu R., Ibim S.E., Maffulli N. (2020). Umbilical cord-derived Wharton’s jelly for regenerative medicine applications. J. Orthop. Surg. Res..

[4] Hwang H.S., Kim H.A. (2015). Chondrocyte Apoptosis in the Pathogenesis of Osteoarthritis. Int. J. Mol. Sci..

[5] Arden N., Nevitt M.C. (2006). Osteoarthritis: Epidemiology. Best Pract. Res. Clin. Rheumatol..

[6] Hopman W.M., Harrison M.B., Coo H., Friedberg E., Buchanan M., VenDenKerkhof E.G. (2009). Associations between chronic disease, age and physical and mental health status. Chronic Dis. Can..

[7] Temple-Wong M.M., Ren S., Quach P., Hansen B.C., Chen A.C., Hasegawa A., D’Lima D.D., Koziol J., Masuda K., Lotz M.K. (2016). Hyaluronan concentration and size distribution in human knee synovial fluid: Variations with age and cartilage degeneration. Arthritis Res. Ther..

[8] Grassel S., Muschter D. (2020). Recent advances in the treatment of osteoarthritis. F1000Res.

[9] Kompel A.J., Roemer F.W., Murakami A.m., Diaz L.E., Crema M.D., Guermazi A. (2019). Intra-articular Corticosteroid Injections in the Hip and Knee: Perhaps Not as Safe as We Thought?. Radiology.

[10] Bedard N.A., DeMik D.E., Glass N.A., Burnett R.A., Bozic K.J., Callaghan J.J. (2018). Impact of Clinical Practice Guidelines on Use of Intra-Articular Hyaluronic Acid and Corticosteroid Injections for Knee Osteoarthritis. J. Bone Joint Surg. Am..

[11] Bhattacharjee S., Wallace S., Luu H.H., Shi L.L., Lee M.J., Chen A.F. (2021). Do We Need to Wait 3 Months After Corticosteroid Injections to Reduce the Risk of Infection After Total Knee Arthroplasty?. J. Am. Acad. Orthop. Surg..

[12] Gupta A., Cady C., Fauser A.M., Rodriguez H.C., Mistovich R.J., Potty A.G.R., Maffulli N. (2020). Cell-free Stem Cell-Derived Extract Formulation for Regenerative Medicine Applications. Int. J. Mol. Sci..

[13] Fabre H., Ducret M., Degoul O., Rodriguez J., Perrier-Groult E., Aubert-Foucher E., Pasdeloup M., Auxenfans C., McGuckin C., Forraz N. (2019). Characterization of Different Sources of Human MSCs Expanded in Serum-Free Conditions with Quantification of Chondrogenic Induction in 3D. Stem Cells Int..

[14] Collins N.J., Prinsen C.A., Christensen R., Bartels E.M., Terwee C.B., Roos E.M. (2016). Knee Injury and Osteoarthritis Outcome Score (KOOS): Systematic review and meta-analysis of measurement properties. Osteoarthr. Cartilage.

[15] Bolognese J.A., Schnitzer T.J., Ehrich E.W. (2003). Response relationship of VAS and Likert scales in osteoarthritis efficacy measurement. Osteoarthr. Cartil..

[16] Kon E., Engebretsen L., Verdonk P., Nehrer S., Filardo G. (2020). Autologous Protein Solution Injection for the Treatment of Knee Osteoarthritis: 3-Year Results. Am. J. Sports Med..

[17] Gupta A., Maffulli N., Rodriguez H.C., Lee C.E., Levy H.J., El-Amin S.F. (2021). Umbilical cord-derived Wharton’s jelly for treatment of knee osteoarthritis: Study protocol for a non-randomized, open-label, multi-center trial. J. Orthop. Surg. Res..

[18] Gupta A., Maffulli N., Rodriguez H.C., Carson E.W., Bascharon R.A., Delfino K., Levy H.J., El-Amin S.F. (2021). Safety and efficacy of umbilical cord-derived Wharton’s Jelly compared to hyaluronic acid and saline for knee osteoarthritis: Study protocol for a randomized, controlled, single-blind, multi-center trial. J. Orthop. Surg. Res..

